# MVB-12, a Fourth Subunit of Metazoan ESCRT-I, Functions in Receptor Downregulation

**DOI:** 10.1371/journal.pone.0000956

**Published:** 2007-09-26

**Authors:** Anjon Audhya, Ian X. McLeod, John R. Yates, Karen Oegema

**Affiliations:** 1 Ludwig Institute for Cancer Research, Department of Cellular and Molecular Medicine, University of California San Diego, La Jolla, California, United States of America; 2 Department of Cell Biology, The Scripps Research Institute, La Jolla, California, United States of America; University of Geveva, Switzerland

## Abstract

After ligand binding and endocytosis, cell surface receptors can continue to signal from endosomal compartments until sequestered from the cytoplasm. An important mechanism for receptor downregulation *in vivo* is via the inward budding of receptors into intralumenal vesicles to form specialized endosomes called multivesicular bodies (MVBs) that subsequently fuse with lysosomes, degrading their cargo. This process requires four heterooligomeric protein complexes collectively termed the ESCRT machinery. In yeast, ESCRT-I is a heterotetrameric complex comprised of three conserved subunits and a fourth subunit for which identifiable metazoan homologs were lacking. Using *C. elegans*, we identify MVB-12, a fourth metazoan ESCRT-I subunit. Depletion of MVB-12 slows the kinetics of receptor downregulation *in vivo*, but to a lesser extent than inhibition of other ESCRT-I subunits. Consistent with these findings, targeting of MVB-12 to membranes requires the other ESCRT-I subunits, but MVB-12 is not required to target the remaining ESCRT-I components. Both endogenous and recombinant ESCRT-I are stable complexes with a 1:1:1:1 subunit stoichiometry. MVB-12 has two human homologs that co-localize and co-immunoprecipitate with the ESCRT-I component TSG101. Thus, MVB-12 is a conserved core component of metazoan ESCRT-I that regulates its activity during MVB biogenesis.

## Introduction

The binding of mitogenic growth factors to cell surface receptors in eukaryotic cells initiates signaling cascades that influence fundamental processes including cell survival, proliferation, differentiation, metabolism, and migration [1], [2]. Constitutive signaling can result in a variety of developmental abnormalities and tumorigenesis [3], indicating that in addition to receptor activation, cellular homeostasis also requires receptor downregulation and degradation. Although ligand binding leads to receptor endocytosis, signaling can continue from endosomal compartments until receptors are sequestered from the cytoplasm [4]–[7]. An important mechanism for downregulation therefore occurs via the inward budding of receptors on endosomal membranes to form intralumenal vesicles in specialized endosomes called multivesicular bodies (MVBs) that subsequently fuse with lysosomes, degrading their cargo [5]. This process requires four heterooligomeric protein complexes (ESCRT-0, -I, -II, -III), collectively termed the ESCRT machinery [8], [9], that facilitate the trafficking of mono-ubiquitylated proteins, the major substrates of the MVB pathway [5], [8], [10]. The subsequent release of ESCRT-I, -II, and -III from membranes requires the AAA-type ATPase Vps4, which catalyzes the recycling of ESCRT components for subsequent rounds of cargo selection and sorting [11]–[13]. In addition to their role in receptor downregulation, many components of the ESCRT machinery have been implicated in the budding of retroviruses such as HIV from the plasma membrane, a process that is topologically similar to the budding of endosomal membranes away from the cytoplasm to form intralumenal vesicles [14], [15].

ESCRT-I is a core component of the ESCRT machinery that functions both in the MVB pathway and retroviral budding. In the MVB pathway, ESCRT-I functions early, along with ESCRT-0 and -II, in the recognition and sorting of mono-ubiquitylated cargo and the formation of intralumenal vesicles. In budding yeast, ESCRT-I was originally characterized as a heterotrimeric complex containing three proteins Vps23p, Vps28p, and Vps37p, that all have clearly conserved metazoan orthologs [16]. Disruption of any of these broadly conserved ESCRT-I components results in a severe phenotype in which morphologically normal MVBs fail to form. Recently, a fourth yeast ESCRT-I subunit, Mvb12p, whose deletion results in a less severe phenotype characterized by a partial sorting defect was described [17]–[20]. Unlike other ESCRT-I components, Mvb12p lacks clear homologs in metazoans. However, both yeast and mammalian ESCRT-I exhibit similar sizing profiles by gel filtration chromatography [16], [20], [21], suggesting that metazoans may also possess a fourth subunit.

Here, we use *C. elegans* to identify and functionally characterize a new subunit of metazoan ESCRT-I. In addition to the three conserved subunits (TSG-101, VPS-28, and VPS-37), tandem affinity purification of VPS-37 revealed the presence of a fourth *C. elegans* ESCRT-I subunit that we name MVB-12. MVB-12 is conserved among metazoans, but is ∼3 fold larger and bears no clear sequence similarity to the yeast protein Mvb12p. Hydrodynamic analysis of endogenous and recombinant ESCRT-I reveals that both are stable heterotetrameric complexes, with a native molecular weight of ∼125 kD, reflecting a 1∶1∶1∶1 association of the four subunits. Depletion of MVB-12 slows the kinetics of cell surface receptor downregulation, consistent with a function in ESCRT-mediated MVB sorting. We also identify two human homologs of MVB-12, MVB12A and MVB12B, that both associate with human ESCRT-I *in vivo*. Together, our data indicate that metazoans, like yeast, possess a fourth subunit that regulates ESCRT-I function.

## Results

### ESCRT-I functions in the downregulation of cell surface proteins in C. elegans

**Figure 1:**
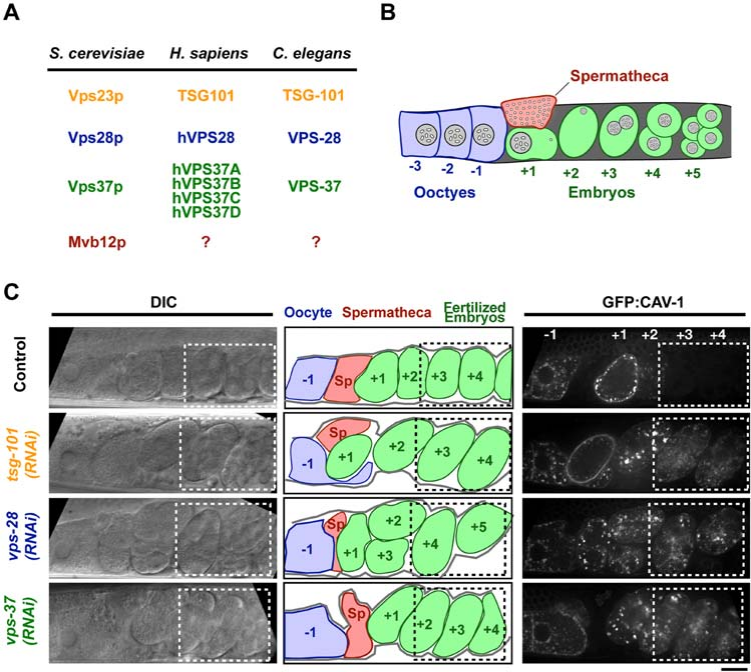
ESCRT-I components mediate degradation of the cell surface protein GFP:CAV-1 after its internalization. (A) Table listing the known subunits of ESCRT-I in *S. cerevisiae, H. sapiens* and *C. elegans*. (B) Schematic of the reproductive system of the adult *C. elegans* hermaphrodite. Oocytes (*blue*) aligned in a row are fertilized in a sequential conveyor belt-like fashion with one ovulation event occurring ∼every 20 minutes [25]. Pre-fertilization oocytes are numbered -1, -2, etc. with respect to their position relative to the spermatheca. Similarly, fertilized embryos (*green*), which are at progressively later stages in development as their distance from the spermatheca increases, are numbered +1, +2, +3, etc. (C) Spinning disk confocal optics were used to image anesthetized control (n = 14), *tsg-101(RNAi)* (n = 15), *vps-28(RNAi)* (n = 11), and *vps-37(RNAi)* (n = 10) adult hermaphrodites expressing GFP:CAV-1. Both differential interference contrast (DIC, *left*) and fluorescence (*right*) images are shown. Schematics in the center column are traces of the DIC images in which the location of the spermatheca (*red*), the unfertilized oocytes (*blue*) and the embryos (*green*) are highlighted. Boxes indicate the presence of embryos at or beyond the two-cell stage, where GFP:CAV-1 is normally absent. Scale bar is 25 µm.

In *C. elegans*, oocyte ovulation and fertilization is accompanied by a series of membrane remodeling events [22] that include the internalization and subsequent degradation of several cell surface markers including the LDL-like yolk receptor RME-2 and CAV-1, a member of the caveolin protein family [23], [24]. The reproducible kinetics of these events with respect to ovulation makes this an excellent system to define the molecular components necessary for the downregulation and subsequent degradation of cell surface proteins. To study the contribution of the ESCRT machinery to the downregulation of cell surface proteins in *C. elegans*, we began by examining the role of ESCRT-I, a heterooligomeric complex required for the internalization of cell surface proteins into intralumenal vesicles to form specialized endosomes called multivesicular bodies (MVBs) that fuse with lysosomes to degrade their cargo. Blast searches identified *C. elegans* homologs for each of the three components of the ESCRT-I complex previously shown to be conserved between yeast and humans (Fig. 1A). To characterize the role of ESCRT-I in the dynamics of cell surface proteins during the oocyte to embryo transition, we began by analyzing GFP:CAV-1. In control oocytes prior to fertilization, GFP:CAV-1 is concentrated in intracellular vesicles and large ring-like cytoplasmic structures as well as localizing weakly to the plasma membrane (Fig. 1C, control, -1 oocyte). Immediately after oocytes pass through the spermatheca and are fertilized, the amount of GFP:CAV-1 on the cell surface rapidly increases, followed by its internalization and degradation [23]. Since, adult *C. elegans* hermaphrodites ovulate approximately every 20 minutes [25], examination of the string of newly fertilized embryos present in an adult worm provides a convenient means of monitoring the timecourse of the changes in the GFP:CAV-1 distribution. Newly fertilized embryos exhibited bright GFP:CAV-1 fluorescence, initially at the cell surface and subsequently on internal membranes (Fig. 1C, control, +1 embryo), but embryos beyond the 2-cell stage, approximately 90 minutes post fertilization, lacked visible fluorescence (Fig 1C, +3 and +4 embryos). Individual depletions of the three conserved ESCRT-I components did not affect the post-fertilization increase in the amount of GFP:CAV-1 on the cell surface or its subsequent re-internalization. However, embryos depleted of each component exhibited a substantial delay in the degradation of internalized GFP:CAV-1, which remained on internal membranes well beyond the two cell stage (Fig. 1C). We also observed similar inhibition of the degradation of internalized RME-2:GFP (described in detail in Fig. 4 below). We conclude that the *C. elegans* ESCRT-I complex has a conserved role in the degradation of internalized cell surface proteins that can be conveniently visualized by monitoring the fate of proteins normally targeted for degradation following fertilization.

### Identification of MVB-12, a fourth subunit of C. elegans ESCRT-I

**Figure 2:**
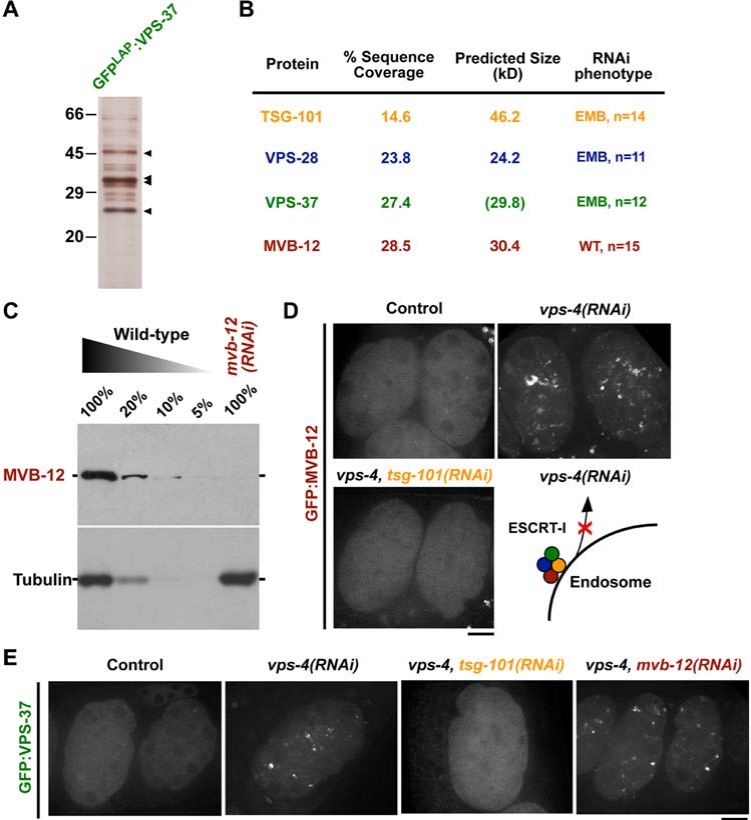
Identification of a fourth *C. elegans* ESCRT-I subunit. (A) A fusion of VPS-37 with a GFP containing tandem affinity purification tag (GFP^LAP^:VPS-37) was isolated from embryo extracts by immunoprecipitation with anti-GFP antibodies. After protease cleavage to remove the GFP tag, proteins were re-isolated on S-protein agarose and eluted. A silver stained gel of the eluted proteins is shown. Arrowheads indicate the predicted mobility of the proteins identified by mass spectrometry. (B) Table summarizing the percent sequence coverage, predicted molecular weight, and RNAi phenotype for each of the four specific proteins in the eluate identified by solution mass spectrometry. For VPS-37, the predicted size including the additional mass of the affinity purification tag is shown in parentheses. In addition to the four proteins shown, a common contaminant (LEV-11) was also identified by mass spectrometry, but is not included in the table. (C) Western blots of extracts prepared from wild type worms or worms specifically depleted of MVB-12 by RNAi. Serial dilutions of extract prepared from untreated worms were loaded to quantify depletion levels. (D) Spinning disk confocal optics were used to image GFP:MVB-12 in control (n = 15), *vps-4(RNAi)* (n = 13), and *vps-4, tsg-101(RNAi)* (n = 15) embryos *in utero*. Schematic illustrates the effect of depleting VPS-4, the AAA-ATPase required to recycle endosome-associated ESCRT-I, returning it to the cytoplasm (*lower right*). Scale bar is 10 µm. (E) Spinning disk confocal optics were used to image GFP:VPS-37 in control (n = 10), *vps-4(RNAi)* (n = 8), *vps-4, tsg-101(RNAi)* (n = 9), and *vps-4, mvb-12(RNAi)* (n = 9) embryos *in utero*. Scale bar is 10 µm.

Recent work in budding yeast identified a fourth ESCRT-I subunit, Mvb12p, for which no metazoan orthologs have yet been identified (Fig. 1A; [17]–[20]). To determine whether *C. elegans* ESCRT-I also possesses an additional subunit, we purified the complex from embryos stably expressing a tandem affinity-tagged form of VPS-37 (Fig. 2A). Analysis of the eluted proteins by mass spectrometry identified each of the known ESCRT-I subunits and one additional protein, which we will refer to as MVB-12, at relatively high sequence coverage (Fig. 2B). In contrast to RNAi-mediated depletion of the other ESCRT-I subunits, which leads to penetrant embryonic lethality, depletion of MVB-12 to less than 5% of endogenous levels (Fig. 2C) failed to affect embryo viability (Fig. 2B), suggesting that MVB-12 is a non-essential component of ESCRT-I. Consistent with this finding, worms homozygous for a deletion allele of *mvb-12, tm2949*, encoding only the first 83 out of 277 amino acids, are viable.

In mammalian cells, ESCRT-I subunits are primarily cytosolic, being only transiently recruited to endosomal membranes followed by rapid release that requires the AAA-type ATPase hVPS4 [11]–[13]. Consistent with this, GFP fusions with VPS-37 and MVB-12 were primarily cytoplasmic in developing oocytes and embryos, and were only occasionally found to associate with a small population of intracellular punctate structures (Fig. 2D, E). Upon depletion of the *C. elegans* Vps4 homolog (VPS-4), however, the GFP fusions with both MVB-12 and VPS-37 strongly accumulated on endosomal membranes, consistent with a requirement for VPS-4 in their disassociation (Fig. 2D, E).

The three broadly conserved components of ESCRT-I are interdependent for their localization to endosomal membranes [21]. In yeast, Mvb12p requires other ESCRT-I components for its endosomal localization [17]–[20]. To test whether MVB-12 requires the presence of other ESCRT-I subunits to associate with membranes in *C. elegans*, we compared the distribution of GFP:MVB-12 in embryos co-depleted of TSG-101 and VPS-4 to that in embryos depleted of VPS-4 alone. In contrast to the dramatic accumulation of GFP:MVB-12 on membranes in VPS-4 depleted embryos, GFP:MVB-12 remained cytoplasmic in embryos that were simultaneously depleted of TSG-101 (Fig. 2D). Similarly, a GFP fusion with VPS-37 also remained cytoplasmic in embryos co-depleted of TSG-101 and VPS-4 (Fig. 2E). In contrast, simultaneous depletion of MVB-12 with VPS-4 failed to prevent the accumulation of GFP:VPS-37 on membranes (Fig. 2E). Together, these data indicate that MVB-12 requires other ESCRT-I subunits for membrane association, but is itself dispensable for targeting of the remaining ESCRT-I subunits.

### ESCRT-I subunits form a 1∶1∶1∶1 complex in vivo and in vitro

Analysis of ESCRT-I in extracts of yeast and human cells fractionated by gel filtration chromatography have shown that ESCRT-I migrates at a position corresponding to a globular complex of between 300–450 kDa, raising the possibility that ESCRT-I oligomerizes in extracts [17]–[21]. More recently however, characterization of the yeast ESCRT-I complex by sedimentation equilibrium centrifugation indicated that it is a heterotetramer of four subunits in a 1∶1∶1∶1 ratio [20]. To determine whether ESCRT-I oligomerizes in extracts, or whether it simply has an elongated shape that causes it to appear large on gel filtration when compared to globular standards, we compared the hydrodynamic properties of recombinant *C. elegans* ESCRT-I to those of the complex present in extracts. Gel filtration chromatography and sucrose density gradients were used to measure the Stokes radii and Sedimentation Values (S-values), respectively, of the native and recombinant complexes, and these values were combined [26] to obtain an accurate estimate of the native molecular weight for each complex.

**Figure 3:**
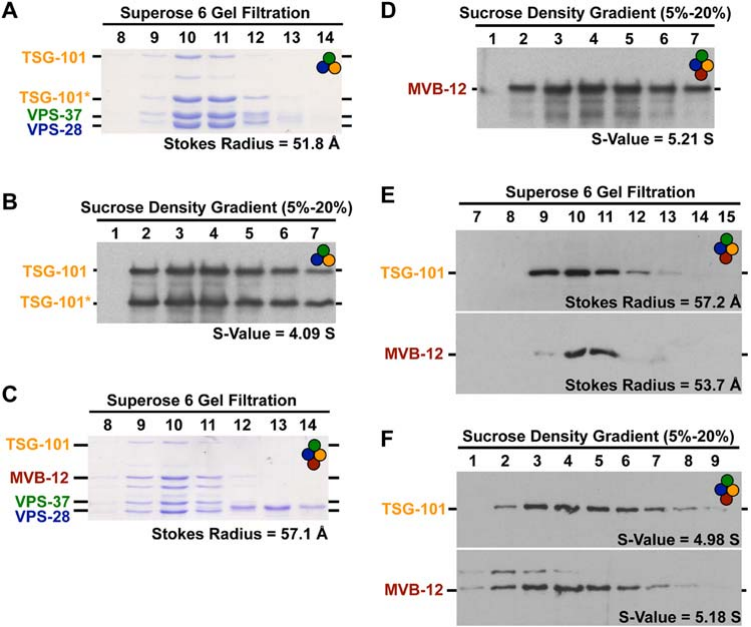
ESCRT-I is a 1∶1∶1∶1 heterotetrameric complex both *in vivo* and *in vitro.* The results presented in each panel are representative of three individual experiments performed. Schematics in the upper right corner of each panel indicate the ESCRT-I components present in each experiment. (A) TSG-101:6xHIS, VPS-28, and VPS-37 were co-expressed and purified from *E. coli* extracts using nickel resin. A Coomassie stained gel of the peak elution fractions after fractionation of the recombinant complex on a Superose 6 gel filtration column is shown. The intensities of each band were measured to identify the peak, and a Stokes radius was calculated for each protein based on comparison with the elution profiles of known standards. The asterisk highlights a breakdown product of TSG-101. (B) The recombinant complex described in A, was fractionated on a 5–20% sucrose gradient. A western blot of the fractions for TSG-101 is shown. The asterisk highlights a breakdown product of TSG-101. The intensities of each band were measured to identify the peak, and S-values were calculated for each protein based on the location of known standards run on a parallel gradient. (C) TSG-101:6×HIS, VPS-28, VPS-37 and MVB-12 were co-expressed and purified from *E. coli* extracts using nickel resin. A Coomassie stained gel of the peak elution fractions after fractionation of the recombinant complex on a Superose 6 gel filtration column is shown. The Stokes radius of the complex, calculated as described for A, is indicated. (D) The recombinant complex described in C, was fractionated on a 5–20% sucrose gradient. A western blot of the fractions for MVB-12 is shown. The S-value of the complex, calculated as described in B is indicated. (E) Western blots of embryo extracts fractionated on a Superose 6 gel filtration column. The peaks corresponding to TSG-101 and MVB-12 are largely overlapping. The Stokes radius of the complex, calculated as described for A, is indicated. (F) Western blots of embryo extracts after fractionation on a 5–20% sucrose density gradient. TSG-101 and MVB-12 co-fractionate, both migrating with an S-value close to 5.0. The S-value of the complex, calculated as described in B is shown.

We began by analyzing recombinant complexes purified after co-expression of multiple ESCRT-I subunits from polycistronic vectors in bacteria. A 6xHis tag fused to the C-terminus of TSG-101 was added to allow complex purification by nickel affinity chromatography. Co-expression of the *C. elegans* homologs of the three previously described metazoan ESCRT-I subunits (TSG-101, VPS-28, and VPS-37) yielded a single complex with an estimated native molecular weight of ∼88 kDa, consistent with a 1∶1∶1 heterotrimeric complex (Stokes radius = 51 Å, S-value = 4.1 S; Fig. 3A, B). Addition of MVB-12 to the polycistronic expression system resulted in a slightly larger complex with an estimated native molecular weight of 125 kDa (Stokes radius = 57.1 Å, S-value = 5.21 S; Fig. 3C, D), nearly identical to the predicted molecular weight of ∼126 kDa for a 1∶1∶1∶1 heterotetrameric complex of the four subunits, indicating that addition of MVB-12 does not cause the complex to oligomerize.

On western blots of embryo extracts fractioned by gel filtration, TSG-101 and MVB-12 peaked at similar positions, corresponding to Stokes radii of ∼57 Å and 54 Å, respectively (Fig. 3E). The Stokes radius of *C. elegans* ESCRT-I is therefore similar to that of the heterotetrameric recombinant complex, and that previously measured for yeast and human ESCRT-I [20], [21]. TSG-101 and MVB-12 also co-migrated on sucrose density gradients, with S-values of ∼5.0 S and 5.2 S, respectively (Fig. 3F). Together these measurements indicate that the *C. elegans* ESCRT-I in extracts has a native molecular weight of ∼119 kDa, very similar to the estimated 125 kDa molecular weight of the recombinant heterotetrameric complex. We conclude that ESCRT-I is comprised of 4 subunits that form a 1∶1∶1∶1 complex both *in vivo* and *in vitro*.

### Depletion of MVB-12 slows the kinetics of receptor downregulation but to a lesser extent than depletion of other ESCRT-I components

**Figure 4:**
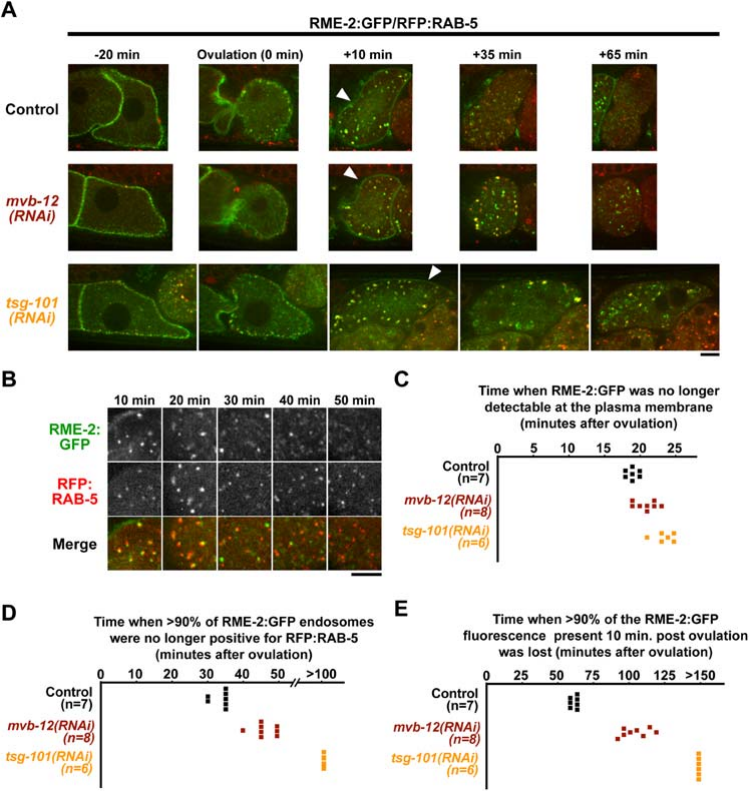
Depletion of MVB-12 slows the degradation of internalized RME-2, but to a lesser extent than inhibition of the ESCRT-I component TSG-101. (A) Spinning disk confocal optics were used to image control (n = 7), *mvb-12(RNAi)* (n = 8), and *tsg-101(RNAi)* (n = 6) embryos co-expressing RME-2:GFP and RFP:RAB-5 *in utero*. Representative sections are shown. Times are in minutes relative to oocyte ovulation. Arrowheads highlight the transient accumulation of RME-2:GFP on the cell surface at the 10 min timepoint. Scale bar is 10 µm. (B) Higher magnification (2x) views of a portion of the control images acquired in (A). At early timepoints after RME-2:GFP internalization, the GFP signal co-localizes with RFP:RAB-5. At later timepoints, both the extent to which the localization of RME-2:GFP is co-incident with that of RFP:RAB-5 and the overall intensity of the RME-2:GFP signal are reduced. Scale bar is 5 µm. (C) The distribution of the time (in minutes after ovulation) when the RME-2:GFP signal was no longer detectable at the cell surface is shown for control, *mvb-12(RNAi)*, and *tsg-101(RNAi)* embryos. (D) The distribution of the time (in minutes after ovulation) when >90% of RME-2:GFP containing endosomes were no longer positive for RFP:RAB-5 is shown for control, *mvb-12(RNAi)*, and *tsg-101(RNAi)* embryos. GFP-positive and RFP-positive endosomes with signals above the cytoplasmic background were identified using individual thresholds. At 1 min intervals, each GFP-labeled endosome was examined for RFP signal. The center of the first 5 min time interval over which less than 10% of the total GFP-positive endosomes also had a detectable RFP signal is plotted for each embryo. (E) The distribution of the time (in minutes after ovulation) when >90% of the RME-2:GFP fluorescence present 10 minutes post ovulation had been lost is shown for control, *mvb-12(RNAi)*, and *tsg-101(RNAi)* embryos. The total integrated RME-2:GFP fluorescence intensity was measured in a 10 µm×10 µm box for each timepoint and the time when this value fell below 10% of the total fluorescence in an identical box at the 10 min timepoint in the same embryo is plotted. Background integrated fluorescence intensity measured in an identical box in older (>50 cell stage) control embryos was subtracted before calculating the percentage of fluorescence remaining.

Although MVB-12 forms a stoichiometric complex with the other three ESCRT-I subunits, depletion of MVB-12, unlike depletion of other members of the complex, does not lead to penentrant embryonic lethality (Fig. 2B). Since depletion of the other three ESCRT-I subunits inhibits the degradation of internalized cell surface proteins, we wanted to determine if depletion of MVB-12 also affects this process. To characterize the effects of MVB-12 depletion, we examined the degradation of a GFP fusion with the LDL-like receptor RME-2. Unlike GFP:CAV-1, which is variably degraded between 30 and 90 minutes post-fertilization, the dynamics of RME-2:GFP are highly reproducible. Within 20 min of ovulation in control zygotes, the entire population of RME-2:GFP has been internalized from the plasma membrane, and is observed coincident with RFP:RAB-5 positive early endosomes en route to degradation (Fig. 4A–C). By 35 minutes post ovulation, more than 90% of RME-2:GFP positive endosomes are no longer positive for RFP:RAB-5, indicating that the RME-2:GFP has progressed beyond the early endosomal compartment (Fig. 4D), and after an additional 30 minutes (∼65 minutes post ovulation), more than 90% of the total RME-2:GFP fluorescence present 10 minutes post ovulation (Fig. 4E) has been lost, indicating that the RME-2:GFP has been largely degraded. The internalization of RME-2:GFP from the plasma membrane occurred with relatively normal kinetics following depletion of either TSG-101 or MVB-12, however delays in the ability of RME-2:GFP to progress beyond RFP:RAB-5 positive structures and in its degradation were observed for both conditions. In TSG-101 depleted embryos, RME-2:GFP continued to localize to RFP:RAB-5 positive compartments more than 100 minutes post ovulation (Fig. 4D) and RME-2:GFP fluorescence was still detected more than 150 minutes post ovulation (Fig. 4E). In contrast, depletion of MVB-12 resulted in more moderate defects. In MVB-12 depleted embryos, the progression of RME-2:GFP to a post RFP:RAB-5 compartment was delayed by ∼10 minutes (Fig. 4D) and an intermediate delay in RME-2:GFP degradation was also observed (Fig. 4E). Cumulatively, these results indicate that although MVB-12 is not essential for the degradation of internalized cell surface proteins, it accelerates the kinetics of this process, consistent with a role for MVB-12 in modulating ESCRT-I activity. It is interesting that inhibition of the fourth yeast subunit, Mvb12p, also exhibits a relatively subtle defect in comparison to loss of the other ESCRT-I subunits [17]–[20], exhibiting only a partial defect in MVB sorting. These results suggest that although the fourth yeast protein is ∼3 fold smaller and exhibits no clear sequence homolog with its metazoan counterparts, there may be some commonalities in function between the yeast and metazoan fourth subunits.

### MVB-12 has two human homologs, MVB12A and MVB12B

**Figure 5:**
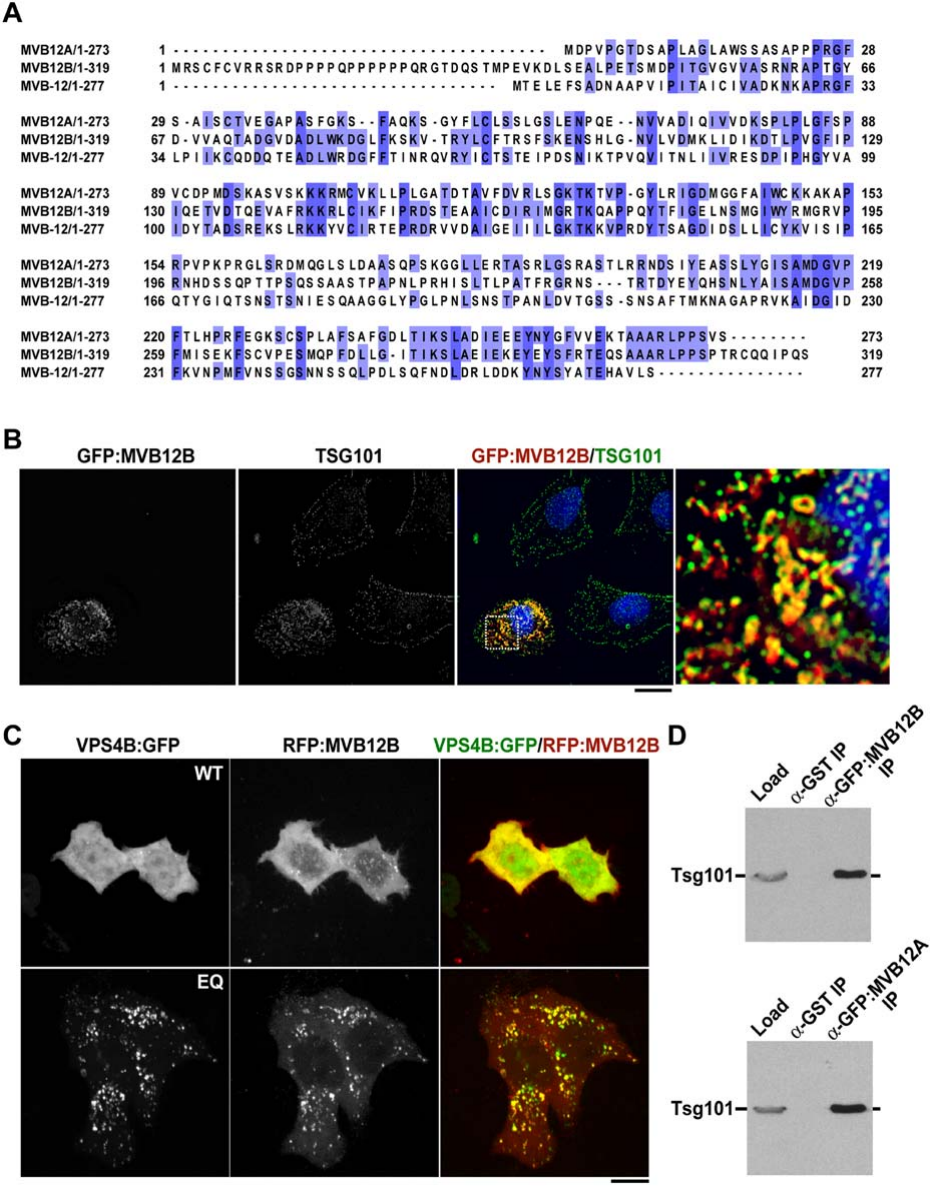
The human MVB-12 homologs, MVB12A and MVB12B, are new components of human ESCRT-I. (A) Sequence alignment of the *C. elegans* protein with MVB12A and MVB12B was performed using ClustalW. Amino acids that are identical between two of the three sequences are highlighted in light blue and between all three sequences in dark blue. (B) HeLa cells transiently transfected with GFP:MVB12B were detergent extracted, fixed and stained with antibodies to GFP and human TSG101 (n = 23). A single section from a representative 3D computationally deconvolved data set is shown. A color overlay of GFP:MVB12B (*red*) and TSG101 (*green*) and a higher magnification (8x) view of the indicated boxed region are also shown. Scale bar is 10 µm. (C) HeLa cells transiently transfected with RFP:MVB12B and either wild type hVPS4B:GFP (n = 13) or mutant hVPS4B^E235Q^:GFP (n = 21) were imaged using spinning disk confocal microscopy. Representative color overlays of RFP:MVB12B (*red*) and hVPS4:GFP (*green*) are also shown. Scale bar is 10 µm. (D) Immunoprecipitations with antibodies to GFP, or GST as a control, were performed on extracts prepared from HeLa cells transfected with either GFP:MVB12A or GFP:MVB12B. Western blots of the starting extracts (load, 10% of total) and the control (10% of total) and GFP (10% of total) immunoprecipitates were probed with antibodies to TSG101.

Although MVB-12 does not exhibit any sequence similarity to the yeast ESCRT-I subunit Mvb12p, we identified a series of metazoan homologs based on its primary sequence, including two human proteins that we will refer to as MVB12A and MVB12B. MVB12A and MVB12B each exhibit approximately 30% identity to MVB-12 (Fig. 5A). Previous studies have shown that overexpression of a single ESCRT-I subunit can titrate other components of the complex and alter endosome morphology [21]. To determine if this was also the case for overexpression of human homologs of the fourth ESCRT-I subunit, we began by examining the effect of overexpression of a GFP fusion with MVB12B, introduced into HeLa cells by transient transfection, on endosome morphology and the localization of TSG101. In cells overexpressing GFP:MVB12B, the distribution of TSG101 was dramatically altered, clustering around the nucleus on membranes decorated with GFP:MVB12B (Fig. 5B). Additionally, the morphology of these membranes was significantly different than TSG101-positive membranes in untransfected cells (Fig. 5B). We conclude that MVB12B can associate with other ESCRT-I components and act in a dominant negative fashion.

The release of ESCRT-I from membranes requires the activity of hVPS4. To test whether MVB12B also requires hVPS4 function, we examined its membrane association in cells expressing wild type hVPS4B or a dominant negative form hVPS4B mutant (E235Q) that blocks ATP hydrolysis and perturbs endosome morphology [12], [13]. While RFP:MVB12B was mostly cytoplasmic in living HeLa cells expressing wild type hVPS4B:GFP, expression of hVPS4B(E235Q):GFP resulted in its accumulation on enlarged ring-like membrane compartments (Fig. 5C). Importantly, RFP:MVB12B and hVPS4B(E235Q):GFP co-localized on these abnormal membrane structures, indicating that release of MVB12B from membranes requires the ATPase activity of hVPS4 (Fig. 5C). Similar results were obtained in experiments using RFP:MVB12A (data not shown).

To test whether MVB12A and/or MVB12B directly interact with ESCRT-I, we immunoprecipitated either GFP:MVB12A or GFP:MVB12B from transfected HeLa cells and probed for the presence of TSG101. We found that TSG101 was dramatically enriched following immunoprecipitation using GFP antibodies in both cases, but was undetectable in control immunoprecipitations using GST antibodies (Fig. 5D). Together, these data indicate that MVB12A and MVB12B are conserved components of human ESCRT-I.

## Discussion

The ESCRT machinery plays a fundamental role in the turnover of membrane bound cargo and functions to terminate receptor signaling in multiple contexts [5]. Additionally, ESCRT-I has been shown to be required for viral budding during HIV infection [14], [15]. Here we use *C. elegans* to identify and characterize a fourth subunit of the metazoan ESCRT-I complex. In addition to the known components TSG-101, VPS-28, and VPS-37, tandem affinity purification of ESCRT-I from embryos followed by mass spectrometry identified MVB-12. Like the other subunits of ESCRT-I, MVB-12 requires TSG-101 to localize to endosomal membranes and the AAA-type ATPase VPS-4 for its subsequent release and recycling. A comparison of the hydrodynamic properties of endogenous and purified recombinant ESCRT-I indicates that MVB-12 is a stable subunit of the complex, which exhibits a 1∶1∶1∶1 subunit stoichiometry. Depletion of MVB-12 perturbs receptor downregulation in *C. elegans*, although not as severely as other ESCRT-I subunits. Based on its primary sequence, we also identified two human homologs of MVB-12, both of which interact with ESCRT-I in HeLa cells and likely define new subunits of human ESCRT-I. Our data suggest that MVB-12 enhances ESCRT-I activity *in vivo* and define a potentially new target for affecting receptor signaling and/or viral budding in human disease.

The metazoan fourth subunits are ∼3 fold larger and bear no clear sequence similarity to the fourth subunit previously identified in yeast. Despite our use of a common nomenclature, the dissimilarities raise the interesting question of why three of the ESCRT-I subunits exhibit broad conservation among eukaryotes, whereas the fourth exhibits a high degree of divergence. Our data suggest that there will likely be commonalities in both the way that the divergent fourth subunits associate with the three conserved subunits and in their function. Several lines of evidence support this hypothesis. First, like deletion of Mvb12p which results in a partial defect in MVB sorting that is relatively subtle in comparison to loss of other ESCRT-I subunits [17]–[20], depletion of MVB-12 delays but does not block receptor downregulation in *C. elegans*, unlike the much more severe phenotype seen following depletion of other ESCRT-I subunits. Second, both the yeast and *C. elegans* ESCRT-I are heterotetrameric complexes with 1∶1∶1∶1 subunit stoichiometries. Third, the behavior of both complexes, as well as human ESCRT-I, during gel filtration chromatography is similar, yielding an apparent molecular weight of 300–450 kD. Based on structural studies, yeast ESCRT-I adopts an elongated conformation that predicts its elution volume on the gel filtration column [20]. The similar gel filtration elution profiles of yeast, *C. elegans* and mammalian ESCRT-I would suggest that the metazoan complex retains a similar conformation. However, a crystal structure of the *C. elegans* or human ESCRT-I complex will be necessary to determine how the larger fourth subunit is incorporated into the ESCRT-I complex.

Despite the similarities, the dramatic divergence of a single component of a conserved trafficking complex between fungi and metazoans raises the possibility that the fourth subunit has evolved unique functions in the two systems. Consistent with this idea, MVB12A was recently identified in a proteomics screen for substrates that are tyrosine-phosphorylated in response to epidermal growth factor (EGF) stimulation, and was shown to function in EGF receptor downregulation [27]. The absence of tyrosine kinases prohibits an analogous event from occurring in yeast. Alternatively, the fourth subunits of ESCRT-I could function as cargo specificity factors, which would likely vary between organisms. Consistent with this idea, loss of Mvb12p in yeast affects the sorting of particular cargos more severely than others [19].

Notably, mammals express two different fourth subunits that only exhibit ∼30% identity at the protein level. Similarly, four different VPS37 isoforms, at least three of which can directly associate with TSG101 [21], [28], have been identified in mammalian cells. The presence of different isoforms for multiple subunits, combined with the 1:1:1:1 stoichiometry of the ESCRT-I complex, indicates that mammalian cells possess a spectrum of ESCRT-I complexes that may function in different contexts or with alternative cargos. Further study will be necessary to uncover the diversity and functional differences between the distinct ESCRT-I complexes that exist in mammalian cells.

## Materials and Methods

### Worm strains and DNA manipulations

The generation of *C. elegans* strains expressing fluorescent fusions with RAB-5 [29], CAV-1 [23], and RME-2 [30] have been described previously. Fluorescent fusions with MVB-12 (C06A6.3) and VPS-37 (CD4.4) were generated by cloning the corresponding unspliced genomic loci into the SpeI site of pIC26 (GFP^LAP^; [31]). Constructs were integrated into DP38 (*unc-119* (*ed3*)) using a PDS-1000/He Biolistic Particle Delivery System as previously described [32]. All strains used in this study are listed in Table S1. *C. elegans* open reading frames corresponding to components of ESCRT-I were amplified from N2 cDNA and cloned into the polycistronic transfer vector pET3aTr, and then into the polycistronic expression construct pST39 [33]. A 6xHIS tag was added to the C-terminus of TSG-101 to enable nickel affinity purification. We thank Barth Grant (Rutgers University, Piscataway, NJ) for providing us with a strain expressing RME-2:GFP.

### RNA-mediated interference and antibody production

dsRNA was prepared as described [34] from templates prepared by using the primers listed in Table S2 to amplify N2 genomic DNA. For complete depletions, L4 hermaphrodites were injected with dsRNA and incubated at 20°C for 40–45 hours, prior to analysis. Antibodies against TSG-101 and MVB-12 were generated by cloning nucleotides 484–948 of C09G12.9 (TSG-101) and 448–834 of C06A6.3 (MVB-12), amplified from a cDNA library, into pGEX6P-1 (Amersham). Purified GST fusion proteins were outsourced for injection into rabbits (Covance). Both antibodies were affinity purified from serum as described [35] by binding to columns of the same antigen after removal of the GST tag by cleavage with Precision protease. Antibodies directed against human TSG101 (Genetex) and GFP have been described previously [31].

### HeLa cell culture and microscopy

HeLa cells were grown in DME supplemented with 10% FBS, penicillin/streptomycin, and L-glutamine (Invitrogen) at 37°C in a humidified atmosphere with 5% CO_2_. Transfections using Effectene (Qiagen) were performed as recommended by the manufacturer. For analysis of fixed HeLa cells, images were acquired using a 100X, 1.35 NA Olympus U-Planapo oil objective lens mounted on a DeltaVision system (Applied Precision) that included an Olympus IX70 microscope equipped with a CoolSnap CCD camera (Roper Scientific). Immunofluorescence of HeLa cells was performed as described [21], using monoclonal α-TSG101 and GFP antibodies at a concentration of 1 µg/ml. For live analysis, worms were anesthetized in 0.1% tricane (Sigma), mounted on agarose pads, and imaged at 20°C on a spinning disc confocal (Nikon Eclipse TE2000-E) microscope equipped with a Nikon 60X, 1.4 NA Planapo oil objective lens and a Hamamatsu Orca-ER CCD camera. Quantification of fluorescence intensity measurements was performed using Metamorph software. HeLa cells were grown on 35-mm glass bottom microwell dishes (MatTek) maintained at 37°C for time-lapse imaging using spinning disk confocal microscopy. The human VPS4 constructs used in our studies were generously provided by Phyllis Hanson (Washington University, St. Louis, MO).

### Mass spectrometry and biochemistry

Adult hermaphrodites expressing GFP^LAP^:VPS-37 were grown synchronously in liquid culture [31] and washed in lysis buffer (50 mM Hepes at pH 7.4, 1 mM EDTA, 1 mM MgCl_2_, 100 mM KCl, and 10% glycerol). Embryos were isolated in buffer containing 0.6 N NaOH and 20% bleach, washed with lysis buffer, and drop frozen in liquid N_2_. Extracts were generated, and VPS-37 interacting proteins isolated as described [31]. Mass spectrometry was performed as described [36], using the most recent version of the predicted *C. elegans* proteins (Wormpep111). Protein expression in BL21 (DE3) *E. coli* was induced with 0.1 mM IPTG for 3 hr at 25°C. Cells corresponding to 3 liters of culture were lysed by sonication in 40 mLs of Lysis Buffer (50 mM Na Phosphate [pH 8.0], 300 mM NaCl, 10 mM imidazole, 0.1% Tween-20, 5 mM β-mercaptoethanol) and centrifuged at 20,000 RPM for 30 min at 4°C. Clarified lysates were incubated in batch with 1 mL of Ni-NTA agarose (Qiagen) for 60 minutes. The resin was subsequently loaded into a column, washed with 200 mL of Lysis Buffer containing 500 mM NaCl, and eluted with buffer containing 50 mM Na Phosphate (pH 7.0), 500 mM NaCl, 250 mM imidazole, and 5 mM β-mercaptoethanol. The eluted protein was exchanged into buffer containing 50 mM Na Phosphate [pH 7.0], 250 mM NaCl, 1 mM EDTA, and 1 mM β-mercaptoethanol using an EconoPac PD10 desalting column (BioRad) and applied to a Superose 6 gel filtration column (GE Healthcare Life Sciences) equilibrated in the same buffer. Prior to gel filtration, embryo extracts were similarly desalted into buffer containing 50 mM Na Phosphate [pH 7.0], 250 mM NaCl, 1 mM EDTA, and 1 mM β-mercaptoethanol. The Stokes radius of each protein complex was calculated from its elution volume based on the elution profiles of standards of known stokes radii (chymotrypsin A, ovalbumin, aldolase, catalase, ferritin and thyroglobulin) run on the same column. Sucrose gradients (5–20%) were poured as previously described [37] and fractionated from the top by hand. Sedimentation values were calculated by comparing the position of the peak with that of standards of known S-value (bovine serum albumin, aldolase, catalase, and thyroglobulin) run on a separate gradient processed in parallel. Immunoblotting of extracts and immunoprecipitations were performed as described [36].

## Supporting Information

Table S1 (0.02 MB DOC)

Table S2 (0.03 MB DOC)

## References

[1] Schlessinger J (2000). Cell signaling by receptor tyrosine kinases.. Cell.

[2] Vivekanand P, Rebay I (2006). Intersection of signal transduction pathways and development.. Annu Rev Genet.

[3] Bache KG, Slagsvold T, Stenmark H (2004). Defective downregulation of receptor tyrosine kinases in cancer.. EMBO J.

[4] Porter AC, Vaillancourt RR (1998). Tyrosine kinase receptor-activated signal transduction pathways which lead to oncogenesis.. Oncogene.

[5] Katzmann DJ, Odorizzi G, Emr SD (2002). Receptor downregulation and multivesicular-body sorting.. Nat Rev Mol Cell Biol.

[6] Sorkin A, von Zastrow M (2002). Signal transduction and endocytosis: close encounters of many kinds.. Nat Rev Mol Cell Biol.

[7] Gonzalez-Gaitan M, Stenmark H (2003). Endocytosis and signaling: a relationship under development.. Cell.

[8] Hurley JH, Emr SD (2006). The Escrt complexes: structure and mechanism of a membrane-trafficking network.. Annu Rev Biophys Biomol Struct.

[9] Williams RL, Urbe S (2007). The emerging shape of the ESCRT machinery.. Nat Rev Mol Cell Biol.

[10] Hicke L, Dunn R (2003). Regulation of membrane protein transport by ubiquitin and ubiquitin-binding proteins.. Annu Rev Cell Dev Biol.

[11] Zhong Q, Chen CF, Chen Y, Chen PL, Lee WH (1997). Identification of cellular TSG101 protein in multiple human breast cancer cell lines.. Cancer Res.

[12] Bishop N, Woodman P (2001). TSG101/mammalian VPS23 and mammalian VPS28 interact directly and are recruited to VPS4-induced endosomes.. J Biol Chem.

[13] Lin Y, Kimpler LA, Naismith TV, Lauer JM, Hanson PI (2005). Interaction of the mammalian endosomal sorting complex required for transport (ESCRT) III protein hSnf7-1 with itself, membranes, and the AAA+ ATPase SKD1.. J Biol Chem.

[14] Demirov DG, Freed EO (2004). Retrovirus budding.. Virus Res.

[15] Morita E, Sundquist WI (2004). Retrovirus budding.. Annu Rev Cell Dev Biol.

[16] Katzmann DJ, Babst M, Emr SD (2001). Ubiquitin-dependent sorting into the multivesicular body pathway requires the function of a conserved endosomal protein sorting complex, ESCRT-I.. Cell.

[17] Chu T, Sun J, Saksena S, Emr SD (2006). New component of ESCRT-I regulates endosomal sorting complex assembly.. J Cell Biol.

[18] Curtiss M, Jones C, Babst M (2007). Efficient cargo sorting by ESCRT-I and the subsequent release of ESCRT-I from multivesicular bodies requires the subunit Mvb12.. Mol Biol Cell.

[19] Oestreich AJ, Davies BA, Payne JA, Katzmann DJ (2007). Mvb12 is a novel member of ESCRT-I involved in cargo selection by the multivesicular body pathway.. Mol Biol Cell.

[20] Kostelansky MS, Schluter C, Tam YY, Lee S, Ghirlando R (2007). Molecular architecture and functional model of the complete yeast ESCRT-I heterotetramer.. Cell.

[21] Bache KG, Slagsvold T, Cabezas A, Rosendal KR, Raiborg C (2004). The growth-regulatory protein HCRP1/hVPS37A is a subunit of mammalian ESCRT-I and mediates receptor down-regulation.. Mol Biol Cell.

[22] Greenstein D, Wormbook (2005). Control of ooctye meiotic maturation and fertilization.. The *C. elegans* Research Community.

[23] Sato K, Sato M, Audhya A, Oegema K, Schweinsberg P (2006). Dynamic regulation of caveolin-1 trafficking in the germ line and embryo of *Caenorhabditis elegans.*. Mol Biol Cell.

[24] Grant B, Hirsh D (1999). Receptor-mediated endocytosis in the *Caenorhabditis elegans* ooctye.. Mol Biol Cell.

[25] McCarter J, Bartlett B, Dang T, Schedl T (1999). On the control of oocyte meiotic maturation and ovulation in *Caenorhabditis elegans*.. Dev Biol.

[26] Siegel LM, Monty KJ (1966). Determination of molecular weights and frictional rations of proteins in impure systems by use of gel filtration and density gradient centrifugation. Application to crude preparations of sulfite and hydroxylamine reductases.. Biochim Biophys Acta.

[27] Konishi H, Tashiro K, Murata Y, Nabeshi H, Yamauchi E (2006). CFBP is a novel tyrosine-phosphorylated protein that might function as a regulator of CIN85/CD2AP.. J Biol Chem.

[28] Eastman SW, Martin-Serrano J, Chung W, Zang T, Bieniasz PD (2005). Identification of human VPS37C, a component of endosomal sorting complex required for transport-I important for viral budding.. J Biol Chem.

[29] Audhya A, Desai A, Oegema K (2007). A role for Rab5 in structuring the endoplasmic reticulum.. J Cell Biol.

[30] Kadandale P, Stewart-Michaelis A, Gordon S, Rubin J, Klancer R (2005). The egg surface LDL-receptor-repeat containing proteins EGG-1 and EGG-2 are required for fertilization in *Caenorhabditis elegans*.. Curr Biol.

[31] Cheeseman IM, Niessen S, Anderson S, Hyndman F, Yates JR (2004). A conserved protein network controls assembly of the outer kinetochore and its ability to sustain tension.. Genes Dev.

[32] Praitis V, Casey E, Collar D, Austin J (2001). Creation of low-copy integrated transgenic lines in *Caenorhabditis elegans*.. Genetics.

[33] Tan S (2001). A modular polycistronic expression system for overexpressing protein complexes in *Escherichia coli*.. Protein Expr Purif.

[34] Oegema K, Desai A, Rybina S, Kirkham M, Hyman AA (2001). Functional analysis of kinetochore assembly in *Caenorhabditis elegans*.. J Cell Biol.

[35] Desai A, Rybina S, Muller-Reichert T, Shevchenko A, Hyman A (2003). KNL-1 directs assembly of the microtubule-binding interface of the kinetochore in C. elegans.. Genes Dev.

[36] Audhya A, Hyndman F, McLeod IX, Maddox AS, Yates JR (2005). A complex containing the Sm protein CAR-1 and the RNA helicase CGH-1 is required for embryonic cytokinesis in *Caenorhabditis elegans*.. J Cell Biol.

[37] Oegema K, Wiese C, Martin O, Milligan R, Iwamatsu A (1999). Characterization of two related *Drosophila* gamma-tubulin complexes that differ in their ability to nucleate microtubules.. J Cell Biol.

