# Supplementary material for: MVB-12, a Fourth Subunit of Metazoan ESCRT-I, Functions in Receptor Downregulation
Source: PLoS One. 2007 Sep 26;2(9):e956. doi: 10.1371/journal.pone.0000956 (PMC1978536; doi:10.1371/journal.pone.0000956)
Supplement: Table S1 — (0.02 MB DOC) [file pone.0000956.s001.doc]

**Table S1. Worm Strains used in this study.**

| Strain # | **Genotype** |
| --- | --- |
| OD176 | unc-119(ed3) III; ltIs103 [pAA212; pie-1/GFPLAP::CAV-1; unc-119 (+)] |
| OD177 | unc-119(ed3) III; ltIs104 [pAA277; pie-1/GFPLAP::VPS-37; unc-119 (+)] |
| OD178 | unc-119(ed3) III; ltIs105 [pAA280; pie-1/GFPLAP::MVB-12; unc-119 (+)] |
| OD179 | unc-119(ed3) III; ltIs79 [pAA196; pie-1/mCherry::RAB-5; unc-119 (+)]; [pie-1/RME-2:GFP] |
