# Supplementary material for: MVB-12, a Fourth Subunit of Metazoan ESCRT-I, Functions in Receptor Downregulation
Source: PLoS One. 2007 Sep 26;2(9):e956. doi: 10.1371/journal.pone.0000956 (PMC1978536; doi:10.1371/journal.pone.0000956)
Supplement: Table S2 — (0.03 MB DOC) [file pone.0000956.s002.doc]

**Table S2.** **dsRNAs used in this study.**

| **RNA#** | **Gene #** | Name | **[ ] mg/ml** | **Oligo #1** | **Oligo #2** | **Template** |
| --- | --- | --- | --- | --- | --- | --- |
| **357** | Y34D9A.10 | *vps-4* | 1.8 | AATTAACCCTCACTAAAGGaaatgttggtttttgagcgg | TAATACGACTCACTATAGGgcatttcggaggttttcgcta | N2 cDNA |
| **344** | C06A6.3 | *mvb-12* | 2.5 | AATTAACCCTCACTAAAGGgtgacggagcttgaattctca | TAATACGACTCACTATAGGgtgaacgcagaattgcttga | N2 cDNA |
| **272** | CD4.4 | *vps-37* | 2.2 | AATTAACCCTCACTAAAGGgcaaaacccgtacgagtcaca | TAATACGACTCACTATAGGgatgacgaccagcagaaggt | N2 cDNA |
| **350** | C09G12.9 | *tsg-101* | 2.1 | AATTAACCCTCACTAAAGGggtttccagacggaaaacgtc | TAATACGACTCACTATAGGctgcattgttgctcggtaga | N2 cDNA |
| **347** | Y87G2A.10 | *vps-28* | 1.9 | AATTAACCCTCACTAAAGGgacaaaacgcgaatttgatgc | TAATACGACTCACTATAGGgtgccttattgaatgcctga | N2 cDNA |
